# Supplementary material for: Patients’ decision-making, experiences and preferences regarding pixantrone treatment in relapsed or refractory diffuse large B-cell lymphoma: study protocol for a longitudinal mixed methods study
Source: BMJ Open. 2019 May 22;9(5):e026505. doi: 10.1136/bmjopen-2018-026505 (PMC6538055; doi:10.1136/bmjopen-2018-026505)
Supplement: Supplementary data [file bmjopen-2018-026505supp001.pdf]

Additional file 1: Topic list

Decision-making related factors

First encounter with treatment with Pixantrone

Initial reaction to treatment availability

The process of weighing of pros and cons

Involvement of others in the decision-making

Trajectory of thoughts and feelings towards initiation of treatment

Actions taken concerning the decision-making

Robustness of final decision

Treatment-related factors

Expectations about treatment benefits

Expectations about treatment side effects

Treatment experiences (including logistics, preferred location (e.g. home or hospital))

Physician related factors

Information provision about Pixantrone treatment (e.g. website)

Physician attitude towards treatment with Pixantrone

Physician-patient communication

Trust in physician

Patient related factors

Disease awareness / knowledge

Treatment history

Quality of life: medical, social and psychological consequences of disease

Family attitude towards treatment

Social support

Spirituality

Practical barriers: finances, transportation etc.
